# Cut-off point of mature oocyte for routine clinical application of rescue IVM: a retrospective cohort study

**DOI:** 10.1186/s13048-023-01294-z

**Published:** 2023-11-22

**Authors:** Jianbo Wei, Zhongyu Luo, Xiyuan Dong, Huizi Jin, Lixia Zhu, Jihui Ai

**Affiliations:** 1 Reproductive Medicine Center, Tongji Hospital, Tongji Medical College, Huazhong University of Science and Technology, 1095 JieFang Avenue, Wuhan, 430030 People’s Republic of China; 2 Department of Gynecology and Obstetrics, Tongji Hospital, Tongji Medical College, Huazhong University of Science and Technology, Wuhan, Hubei China; 3 Department of Obstetrics and Gynecology, Center for Reproductive Medicine, Guangdong Provincial Key Laboratory of Major Obstetric Diseases; Guangdong Provincial Clinical Research Center for Obstetrics and Gynecology; Guangdong-Hong Kong-Macao Greater Bay Area Higher Education Joint Laboratory of Maternal-Fetal Medicine, The Third Affiliated Hospital of Guangzhou Medical University, Guangzhou, China; 4 Key Laboratory for Reproductive Medicine of Guangdong Province, The Third Affiliated Hospital of Guangzhou Medical University, Guangzhou, China

**Keywords:** Rescue in vitro maturation, Mature oocyte, Cut-off point, Cumulative live birth rate, IVF/ICSI outcome, Live birth rate, ART treatment predictors

## Abstract

**Background:**

The rescue in vitro mature(Rescue IVM) technique allows the use of immature oocytes collected in conventional COH to obtain more mature oocytes for fertilization through in vitro maturation. Some studies have shown that Rescue IVM could improve clinical outcomes in patients undergoing IVF/ICSI, but the effectiveness and the indications for the clinical application of this technique remain controversial. It remains to be studied whether Rescue IVM should be universally applied in all conventional IVF/ICSI cycles.

**Method:**

This is a large retrospective cohort study that included a total of 22,135 female patients undergoing their first IVF treatment cycles. The effect of the number of mature oocytes(metaphaseII[MII]) on the cumulative live birth rate was investigated in a population with routine IVF/ICSI first. The receiver operating characteristic curve(ROC) analysis was used to explore the cut-off point of the number of MII affecting CLBR. Secondly, Patients undergoing ICSI with Rescue IVM were included in the analysis with those who underwent ICSI only during the same period, grouped according to the MII cut-off values. Multi-factor binary logistic regression and inverse probability weighting (IPW) were used to investigate whether Rescue IVM influenced the final cumulative live birth rate(CLBR).

**Results:**

The CLBR increased with the number of MIIoocytes (P < 0.001). The ROC analysis showed the cut-off point for the number of MIIoocytes to have a significant effect on CLBR was 9 (sensitivity 0.715, specificity 0.656). Furthermore, 912 patients who underwent ICSI with Rescue IVM were included and compared to those who underwent ICSI only during the same period, and found Rescue IVM significantly increased the number of available MIIoocytes. For patients with MII numbers < 9, Rescue IVM significantly improves their clinical pregnancy rate(55.6% vs. 46.7%, P = 0.001) and CLBR(65.4% vs. 48.1%, P < 0.001), but not for those patients with MII numbers ≥ 9.

**Conclusion:**

This study further clarifies the candidates for the application of Rescue IVM technique: patients with an MII oocytes < 9 in a conventional IVF/ICSI cycle. In contrast, it is not necessary for patients who already have sufficient mature oocytes(≥ 9), to avoid over-medication.

**Supplementary Information:**

The online version contains supplementary material available at 10.1186/s13048-023-01294-z.

## Introduction

In 1978, Robert Edwards et al. collected oocytes by the natural cycle, performed in vitro fertilization and embryo transfer (IVF-ET) and successfully delivered a normal, healthy baby weighing 2700 g [1], in subsequent decades, more and more infertile couples were benefiting from various types of assisted reproductive technology (ART). In China, the total number of babies born through ART was 311,309 in 2016, accounting for 1.69% of all live babies [2]. IVF has become an important routine treatment for infertility. During the development of ART, controlled ovarian hyperstimulation (COH) is an important technology to improve the IVF success rate. COH induces multiple follicle development, and it also greatly improves the probability of pregnancy in each cycle [3]. In addition to conventional IVF/ICSI cycles after ovarian stimulation, Rescue IVM is increasingly becoming a method of increasing the number of available embryos, especially in patients with higher than expected immature oocytes [4]. With the significant progress in cryopreservation techniques such as vitrification [5], the number of euploid embryos for embryo transfer increases, which further affects the overall cumulative live birth rates(CLBR) [6].

Classic IVM is often defined as the in vitro maturation of oocytes retieved from follicles at the GV or MI stage after exposure to exogenous FSH and/or hCG [7]. While the Rescue IVM refers to the collection of immature oocytes from conventional IVF/ICSI cycles and culturing them in vitro to the metaphase II(MII) stage for subsequent fertilization and embryo culture [8]. Rescue IVM can help patients make the most of the immature oocytes that are often discarded in conventional IVF/ICSI cycles to obtain more embryos available for transfer, and to increase live birth rates [9], especially for patients with low functional ovarian reserve, every additional mature oocyte or embryo has considerable potential clinical significance [10]. The reproductive potential and safety of oocytes obtained by Rescue IVM remains controversial [11–14].

The most obvious effect of Rescue IVM is to increase the number of mature oocytes available. However, whether more oocytes means better reproductive outcomes is still debated. In IVF/ICSI treatment, it has been agreed that the number of oocytes retrieved are predictors of pregnancy [15]. In most studies, it is generally accepted that 12–18 is the optimal oocytes range for the highest live birth rate in the fresh IVF/ICSI cycles(fLBR) [16–18]. In terms of CLBR, the common view is that cumulative live birth rates steadily increased with the number of oocytes [19–22], but some studies have also shown that there is a plateau in CLBR growth as the number of oocytes increases, meaning that excess oocytes do not lead to a better CLBR [23, 24]. Meanwhile, studies have shown that the increasing estradiol levels after COH cause the damage of endometrial receptivity and affect the embryo quality, leading to a decrease in CLBR [25, 26].

However, previous studies mainly concentrated on the relationship between the total number of oocytes retrieved and the fresh live birth rate(fLBR) or CLBR, as the total number of oocytes retrieved always reflects the ovarian response [27]. Nevertheless, in the routine IVF/ICSI treatment regimens of most medical institutions, MII oocytes are directly used for fertilization and subsequent embryo culture due to their better fertilization and embryonic development ability, while the immature oocytes(GV or MI) which could have been used by Rescue IVM are often sacrificed. In the application of Rescue IVM, there is no consensus on whether patients who undergo conventional IVF/ICSI can benefit from Rescue IVM, some studies have concluded that the clinical value of IVM is not as good as conventional IVF/ICSI at present [28, 29], but there are also studies suggesting that Rescue IVM improved reproductive outcomes in women undergoing ICSI [30]. These conflicting conclusions bring up a question: for those patients who already have enough mature oocytes, is it necessary for them to undergo Rescue IVM to obtain additional MII oocytes? We conducted this study to investigate the association between the number of MII oocytes and the CLBR in patients undergoing conventional IVF/ICSI, to find the MII oocytes count threshold affecting CLBR, and verify whether Rescue IVM could help these “mature oocyte deficient” patients to achieve better live birth outcomes.

## Materials and methods

### Study Population

This retrospective cohort study collected data from the patients at the Reproductive Center of Tongji Hospital, Tongji Medical College, Huazhong University of Science and Technology, from January 2015 to December 2019 who underwent their first IVF or ICSI cycle, and patients undergoing Rescue IVM from January 2016 to December 2019. The study was approved by the institutional review board of Tongji Hospital. Individual patient data were collected from medical records database of Tongji hospital, including patient demographic characteristics, examination results, treatment process and outcomes. The clinical information had been fully anonymized before analysis.

The exclusion criteria for the study: (1) patients with oocyte donated or oocyte cryopreserved, (2) patients who underwent pre-implantation genetic testing (PGT), (3) patients with remaining frozen embryos without obtaining a live birth, (4) patients with natural cycle, (5) patients who failed to obtain MIIoocytes. Overall, 22,135 female patients were eventually enrolled, 912 of whom were treated with Rescue IVM.

### Clinical protocols

Populations using three main COH regimens were included in the study, including (1) the Depot GnRH-agonist regimen, (2) the Daily GnRH-agonist regimen, and (3) the GnRH-antagonist regimen. The down-regulation methods and ovarian stimulation protocols were performed as described in previous literature [31]. Briefly, the depot GnRH-agonist regimen was administered with 3.75 mg Triptorelin on the first day of the menstrual cycle. 150–225 IU rFSH per day was started 28 days later until the human chorionic gonadotropin (hCG) trigger day. The daily GnRH-agonist regimen was 0.1 mg/d of GnRH-a (Decapeptyl, Ferring, Switzerland, or Diphereline, Ipsen, Australia) subcutaneously from mid-luteal phase until pituitary suppression was reached with gonadotropins (rFSH; Gonal-F, Seron, Switzerland, or Puregon, Organon, The Netherlands) to start ovarian stimulation, while the dose of GnRH-a was reduced to 0.05 mg/d until the administration date of hCG. As for the GnRH-antagonist regimen, recombinant follicle-stimulating hormone (r-FSH) was started on day 2 or 3 of the menstrual cycle when the leading follicle reached an average diameter of 12–14 mm with subcutaneous cetrotide acetate (Cetrotide; Merck Serono, Geneva, Switzerland) at a dose of 0.25 mg/d. When the mean diameter of the 2–3 follicles reached 18 mm, 10,000 IU rhCG (Ovidrel; Merck-Serono, Geneva, Switzerland) was injected to trigger ovulation. Oocytes were obtained by transvaginal ultrasound-guided puncture 36–38 h after hCG injection. The granulosa cells surrounding the oocytes were removed using hyaluronidase (Vitrolife, Sweden) 2 to 4 h after egg collection, and the period of time the oocytes were in was assessed and recorded. ICSI or Rescue IVM combined with ICSI was then performed. For patients undergoing ICSI with Rescue IVM, their immature oocytes were collected and continued to be cultured in G1-plus medium (Vitrolife, Sweden) and checked for oocyte maturity every 6 h until 24 h. MII stage oocytes were used for ICSI fertilization.

### IVF and embryos culture

Depending on the quality of the sperm, fertilization can be performed by IVF or intracytoplasmic sperm injection (ICSI). For rescued IVM-derived MII eggs, ICSI was the preferred method of fertilization in our center. Oocytes were incubated in G-IVF medium (Vitrolife, Sweden) and fertilized 3–4 h after oocyte retieved. Oocytes were checked for fertilization 16–18 h after fertilization and were considered successfully inseminated if zygotes with two pronuclei (2PN) were visible. On day 3 after retrieval of the oocytes, one or two embryos of the best quality are transferred. Embryos are scored according to the number of blastomeres, the homogeneity of the blastomeres and the number of embryonic fragments at the speed of embryonic development on day 3 [32]. Blastocysts were also evaluated with 3 main morphological parameters: the stage of development, inner cell mass(ICM), andtrophoectoderm(TE) [33].

### Embryo vitrification and frozen-embryo transfer

The remaining high-quality day 3 embryos or blastocysts were cryopreserved for subsequent FET cycles. Details of embryo cryopreservation and frozen-thawed embryo transfer protocols have been described previously [34]. Our center used endometrial preparation for FET cycles with natural cycles (NC) or artificial cycles (AC). FET is performed 3 or 5 days after endometrial transformation, followed by luteal phase support until the 10th week of gestation.

### Main outcome measures

The primary outcome was the CLBR defined as at least one live birth either by fresh embryo transfer or subsequent frozen-thawed cycle in a complete oocyte retrieval cycle. Meanwhile live birth means newborns delivered with at least one of four vital signs: heartbeat, respiration, umbilical cord pulsation, and random muscle contraction. The secondary outcome was live birth rate after fresh cycle.

### Statistical analysis

Patient demographic characteristics, ovarian response, IVF/ICSI treatment characteristics, and pregnancy outcomes were entered into spreadsheets and used for statistical analysis. Continuous variables were expressed as mean ± SD or median (IQR), and categorical variables were expressed as percentages.

The characteristics of the live-birth and non-live-birth populations were described separately. Bar graphs were used to describe the overall trend of mature oocytes count with changes in CLBR and fLBR. Patients were divided into four groups according to mature oocyte count: 1–3(Group A), 4–8 (Group B), 9–14 (Group C) or ≥ 15 (Group D), one-way analysis of variance (ANOVA), Kruskal-Wallis test and chi-square test were performed appropriately for different type of data. Multiple logistic regression models with CLBR as the dependent variable were developed to analyze the relationship between different numbers of MII oocytes and CLBR after inclusion of confounding factors(Logistic regression model results are presented as ORs with SEs and 95% CIs.). The above data were analyzed using SPSS(SPSS Inc, version 23, Chicago, IL, USA) for data analysis. The ROC curve analysis was constructed using MedCalc statistical software (MedCalc version 20.0.26, Mariakerke, Belgium) and the Youden index was used to determine the cut-off value of the total number of eggs retrieved and the number of MII oocytes with respect to the CLBR.

Patients undergoing ICSI with Rescue IVM were included in the analysis with those who underwent ICSI only during the same period, grouped according to the MII cut-off values. The characteristics and treatment outcomes were compared between different treatment groups. When further investigating whether Rescue IVM influenced the final CLBR, we used two probability-adjusted models, multi-factor binary logistic regression and inverse probability weighting (IPW), to eliminate the effect of confounding factors. The results of the models are expressed as aOR with SEs and 95% CIs. All statistics were considered statistically significant when the value of P < 0.05.

### Institutional Review Board Statement

The study was conducted in accordance with the Declaration of Helsinki, and approved by the Institutional Review Board (or Ethics Committee) of Tongji Hospital, Tongji Medical College, Huazhong University of Science and Technology (IRB ID: TJ-C20211013; 2021.10.23).

## Results

### Patient characteristics

Of the final 22,135 female patients included in the analysis, Table 1 shows the patients’ characteristics grouped by ART outcome. 14,694 (66.38%) patients achieved live births in fresh embryo transfer or frozen-thawed cycles while 7,441 (33.62%) patients did not after all embryos were used. In the total population, the average oocyte maturation rate was 88.12% and the patients’ average age was 30.9 years, tubal factors were the main cause of infertility (50.1%), and most patients underwent IVF (68.2%) for fertilization. Women in the live-birth group were younger than the other group on average (29.97 vs. 32.71) and had a lower mean basal FSH (7.37 vs. 8.36). Among the causes of infertility, a higher proportion of the non-live birth group was diagnosed with DOR (3.8% vs. 8.1%). Regarding the number of MII obtained, a higher proportion of the non-live birth group was in group A (1–3) (24.4% vs. 3.5%) and a lower proportion in group D (≥ 15) (8.7% vs. 30.7%).

**Table 1:** Patient characteristics by ART outcome

Characteristic	All patients(n = 22,135)	Live birth(n = 14,694)	Non-live birth(n = 7441)
**Age(years)**	30.9 ± 4.68	29.97 ± 3.87	32.71 ± 5.52
**Duration of infertility (years)**	3.53 ± 2.72	3.34 ± 2.40	3.90 ± 3.23
**Type of infertility, n (%)**			
Primary	14,220(64.2%)	9776(66.5%)	4444(59.7%)
Secondary	7915(35.8%)	4918(33.5%)	2997(40.3%)
**Cause of infertility, n (%)**			
Tubal	11,064(50.1%)	7585(51.6%)	3479(46.8%)
Ovulatory	2266(10.2%)	1807(12.3%)	459(6.2%)
Male	3733(16.8%)	2514(17.1%)	1219(16.4%)
DOR	1161(5.2%)	558(3.8%)	603(8.1%)
Endometriosis	1973(8.9%)	1350(9.2%)	623(8.4%)
Uterine disease	568(2.6%)	347(2.4%)	221(3.0%)
Unexplained	1370(6.1%)	533(3.6%)	837(11.1%)
**BMI (kg/m** ^**2**^ **)**	21.88 ± 2.95	21.80 ± 2.93	22.09 ± 3.02
**Number of mature oocytes, n (%)**			
1–3	2326(10.5%)	508(3.5%)	1818(24.4%)
4–8	6737(30.4%)	3673(25.0%)	3064(41.2%)
9–14	7910(35.7%)	6002(40.8%)	1908(25.6%)
≥15	5162(23.4%)	4511(30.7%)	651(8.7%)
**FSH (mIU/mL)**	7.71 ± 2.78	7.37 ± 2.25	8.36 ± 3.51
**P(ng/ml)**	0.94 ± 0.71	0.95 ± 0.61	0.92 ± 0.90
**Insemination method, n (%)**			
IVF	15,105(68.2%)	10,177(69.3%)	4928(66.2%)
ICSI	7030(31.8%)	4517(30.7%)	2513(33.8%)
**Down-regulation method, n (%)**			
Long GnRH agonist	8797(39.9%)	6620(45.0%)	2207(29.7%)
GnRH-antagonist	5351(24.3%)	2743(18.7%)	2638(35.4%)
Daily GnRH-agonist	7897(35.8%)	5331(36.3%)	2596(34.9%)
**No. of transferred blastocyst, n**	1.01 ± 0.83	1.10 ± 0.80	0.84 ± 0.85

Values are expressed as mean ± SD for continuous variables and n (%) for categorical variables

DOR = diminished ovarian reserve; BMI = body mass index; FSH = follicle-stimulating hormone; P = Progesterone; ICSI = intracytoplasmic sperm injection; GnRH = gonadotropin-releasing hormone

### Association between the number of MII oocytes and LBR/CLBR

**Fig. 1:**
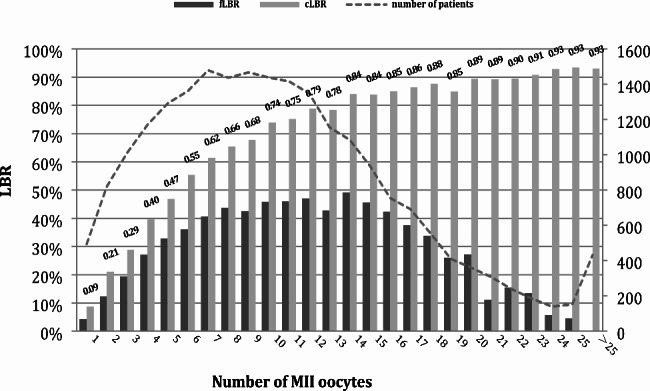
Cumulative and fresh LBRs according to the number of MII oocytes retrieved

Figure 1 shows the fLBR and CLBR outcomes according to the number of MII oocytes. The fLBR initially increased with the increase in the number of MII oocytes until the number of MII reached 8, with the LBR 43.7%, and then reached a plateau (42.6-49.1%) when the number of MII was 9–14, followed by a decrease. While the CLBR increased steadily with the increase in the number of MIIoocytes beyond 25, when the cumulative live birth rate reached 93.0% and there was no obvious plateau period. The folded line represents the number of patients who obtained that number of MII.

To examine the relationship between the number of MII and CLBR in different age groups, we grouped age (< 30, 30–35, > 35 years). The results (Supplemental Fig. 1) showed that although the overall CLBR decreased with increasing age, the CLBR showed a positive trend in relation to the number of MII in the different age subgroups.

The patients were divided into four groups according to the number of MIIoocytes retrieved, Table 2 presents the treatment outcome indicators such as oocyte maturation rate, fertilization rate, blastocyst rate, fLBR and CLBR. The group of patients with more mature oocytes obtained were younger and with a higher CLBR. Interestingly, the oocyte maturation rate, fertilization rate, and blastocyst formation rate were also increased with the number of MIIoocytes, as well as more transferable embryos (p < 0.001). However, there was no significant difference in Cleavage rate(p = 0.994). That seemed to mean that the patients with high MII numbers also had better oocyte quality. Whereas the fLBR was significantly higher in Group C (9–14) than the other three groups (p < 0.001).

**Table 2:** IVF/ICSI outcome characteristics of the different MIIoocytes group

Group	A(1-3 MII) N = 2326,10.5%	B(4-8 MII) N = 6737,30.4%	C(9-14 MII) N = 7910,35.7%	D(≥ 15 MII) N = 5162,23.4%	P-value
**Fresh live birth rate (%)**	318(13.67%)	2462(36.54%)	3597(45.47%)	1510(29.25%)	<0.001^c^
**Cumulative live Birth rate (%)**	508(21.84%)	3673(54.52%)	6002(75.88%)	4511(87.39%)	<0.001^c^
**Age(years)**	34.48 ± 5.69	31.80 ± 4.83	30.14 ± 4.03	29.25 ± 3.65	<0.001^b^
**Oocytes retrieved**	3(2–3)	7(6–9)	13(11–15)	20(18–24)	<0.001^a^
**Maturation rate (%)**	0.849 ± 0.225	0.861 ± 0.154	0.887 ± 0.115	0.914 ± 0.087	<0.001^b^
**Fertilization rate(%)**	0.724 ± 0.342	0.760 ± 0.223	0.783 ± 0.185	0.806 ± 0.164	<0.001^b^
**Cleavage rate(%)**	0.878 ± 0.268	0.9014 ± 0.177	0.9112 ± 0.138	0.9124 ± 0.122	0.994^b^
**Blastocyst rate (%)**	0.370 ± 0.450	0.539 ± 0.366	0.609 ± 0.281	0.652 ± 0.234	<0.001^b^
**Freeze-all rate (%)**	684(29.4%)	1287(19.1%)	1428(18.1%)	2649(51.3%)	<0.001^c^
**Gn duration(days)**	9.46 ± 2.23	10.13 ± 1.96	10.39 ± 1.80	10.57 ± 1.88	<0.001^b^
**Gn dosage(IU)**	2400(1725–3000)	2400(1695–3000)	1950(1405–2550)	1665(1275–2212)	<0.001^a^
**Embryos transferable**	1(0–2)	2(2–3)	4(2–5)	6(4–8)	<0.001^a^

^a,^Kruskal–Wallis test. Values are Median (IQR).

^b,^One-way ANOVA. Values are mean + SD.

^c,^Pearson x^2^ test. Values are number (percentage)

**Fig. 2:**
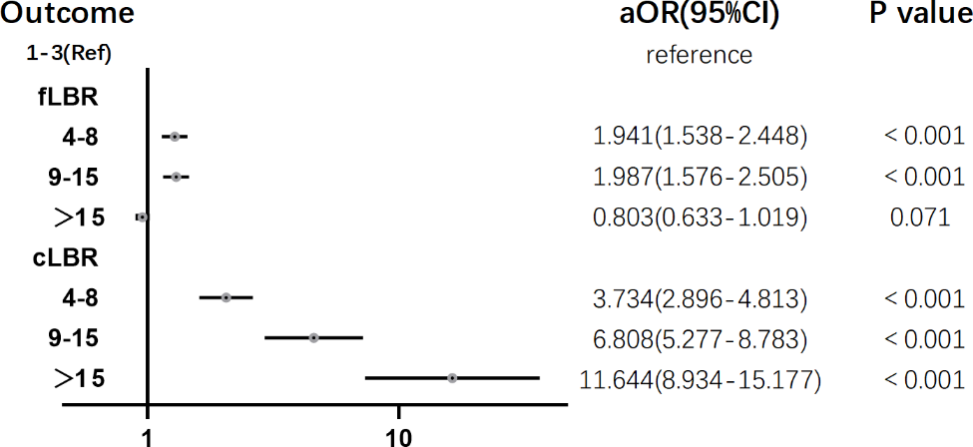
Forest plot of adjusted odds ratio of live birth outcomes

Using Multivariable logistic regression to adjust for confounding factors, Fig. 2 demonstrates the effect of increasing MII number group on LBR after fresh embryo transfer and CLBR after adjustment. For CLBR, the ORs of the MII number 4–8, 9–14, and ≥ 15 groups were 3.734 (2.896–4.813), 6.808 (5.277–8.783), and 11.644 (8.934–15.177) respectively (p < 0.001) compare to the control group(1–3). As for LBR, the ORs of the MII numbers 4–8, 9–14 remained significant compared to the control group, but did not show a statistically significant difference when MII number ≥ 15(P = 0.071).

### The cut-off point of mature oocyte count regarding CLBR

**Fig. 3:**
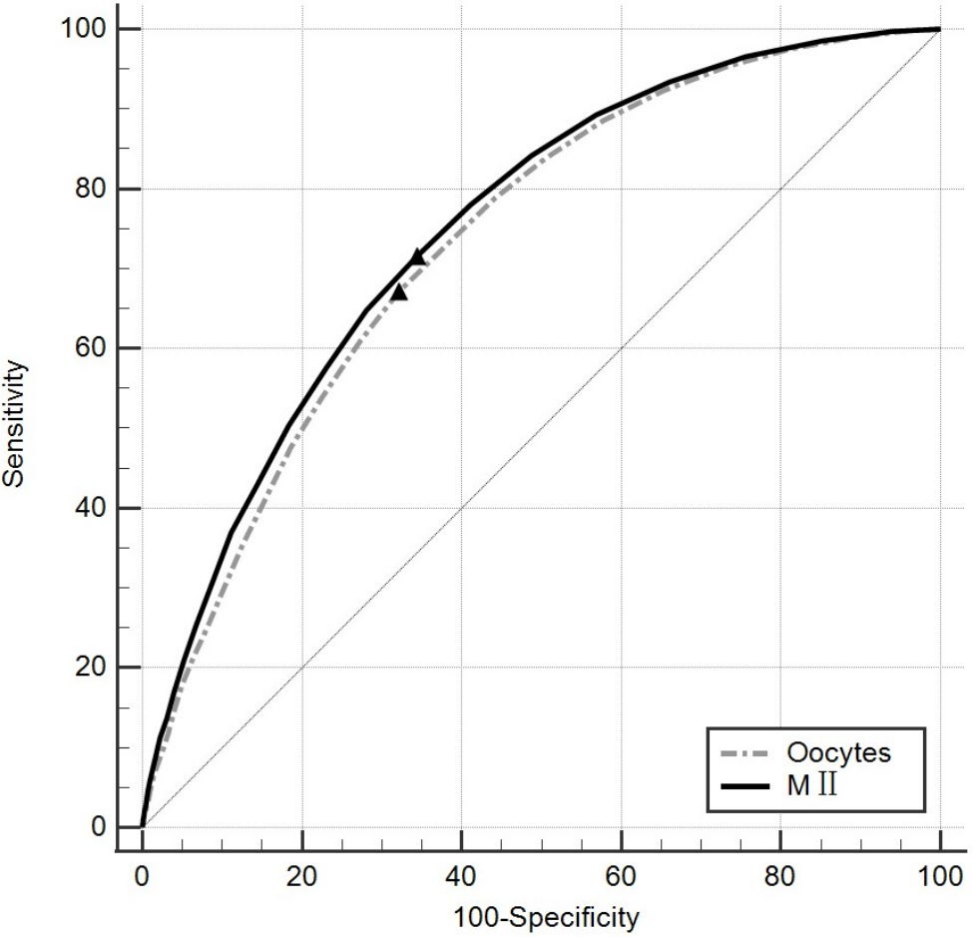
The number of mature oocytes and total oocytes in the prediction of CLBR by receiver operator characteristic curve (ROC)

Figure 3 shows the ROC curve analysis of the number of MIIoocytes and the total number of oocytes with respect to the CLBR, and calculated the cut-off values by Youden index (Supplementary Table 1). The cut-off value for MIIoocytes was 9 (sensitivity 0.7155, specificity 0.6561) and the total number of oocytes was 11 (sensitivity 0.6707, specificity 0.6793). Supplementary Fig. 2 shows the ROC results on fLBR, however, both the total number of eggs retrieved(AUC = 0.522) and the MIIoocytes(AUC = 0.530) are poor predictors of overall fLBR. Moreover, we compare the predictive ability of the number of MIIoocytes and the total number of oocytes in predicting CLBR, we compared their AUC and found that the AUC of MII was greater than the AUC of the total number of eggs retrieved (7.52 vs. 7.36, P < 0.0001) (Supplementary Table 2), suggesting that using MII to predict CLBR has greater sensitivity and accuracy.

In order to verify whether patients with less than 9 MII oocytes could increase the cumulative live birth rate by Rescue IVM, patients were divided by cut-off values into two groups: MII oocytes < 9 and ≥ 9 (before Rescue IVM), and basic patient information (Table 3) and cumulative treatment outcomes (Table 4) were counted separately. We found that in both groups, performing Rescue IVM resulted in a significant increase in the number of MII, and in patients with MII<9, it also resulted in a significant reduction in the proportion of patients whose cycles were canceled due to lack of available embryos compared to conventional ICSI (3.9% vs. 12.5%, P < 0.001). In terms of treatment outcomes, Rescue IVM did not appear to significantly contribute to fresh transfer cycles. However, after considering FET, patients with Rescue IVM had significantly higher rates of implantation, clinical pregnancy and cumulative live births than controls in the < 9 group. However, in patients with MII numbers ≥ 9, Rescue IVM did not significantly contribute to clinical outcomes.

**Table 3:** Patient characteristics of ICSI only and IVM with ICSI patients

	Mature oocytes < 9			Mature oocytes ≥ 9		
	**IVM + ICSI** **(n = 873)**	**ICSI** **(n = 1725)**	**P**	**IVM + ICSI** **(n = 39)**	**ICSI** **(n = 2716)**	**P**
Age (years)	30.0 (27.0 ~ 32.0)	31.0 (28.0 ~ 35.0)	< 0.001	29 (27 ~ 31)	29 (27 ~ 31)	0.905
BMI (kg/m^2^)	21.6 (19.7 ~ 23.7)	21.5 (19.7 ~ 23.9)	0.931	21.0 (19.1 ~ 24.0)	21.2 (19.5 ~ 23.3)	0.745
Infertility duration(years)	3.0 (2.0 ~ 5.0)	3.0 (2.0 ~ 5.0)	0.312	3.0 (2.0 ~ 4.0)	3.0 (2.0 ~ 4.0)	0.725
Infertility type			0.005			0.366
Primary	637(73.0)	1345 (78.0)		30 (76.9)	2240 (82.5)	
Secondary	236 (27.0)	380 (22.0)		9 (23.1)	476 (17.5)	
Infertility causes			< 0.001			< 0.001
Female	135 (15.5)	473 (27.4)		19 (48.7)	535 (19.7)	
Male	432 (49.5)	479 (27.8)		8 (20.5)	1233 (45.4)	
Both	211 (24.2)	696 (40.3)		7 (17.9)	778 (28.6)	
Unknown	95 (10.9)	77 (4.5)		5 (12.8)	170 (6.3)	
Basal FSH (mIU/ml)	7.1 (6.2 ~ 8.2)	7.6 (6.4 ~ 8.9)	< 0.001	6.1 (5.4 ~ 7.1)	6.9 (6.0 ~ 7.9)	0.001
AFC	14 (10 ~ 20)	10.0 (7.0 ~ 15.0)	< 0.001	22.0 (15.0 ~ 24.0)	16.0 (12.0 ~ 22.0)	< 0.001
AMH (ng/ml)	4.8 (2.9 ~ 8.0)	3.0 (1.7 ~ 5.4)	< 0.001	7.5 (5.8 ~ 10.9)	6.1 (3.9 ~ 9.2)	0.002
COH protocol			< 0.001			0.765
GnRH-a	681 (78.0)	1107 (64.2)		35 (89.7)	2366 (87.1)	
GnRH-ant	192 (22.0)	541 (35.8)		4 (10.3)	350 (12.9)	
Gn duration(days)	10.0 (9.0 ~ 12.0)	10.0 (9.0 ~ 11.0)	< 0.001	11 (10 ~ 12)	10 (9 ~ 11)	0.051
Gn dosage(IU)	2197.5 (1650.0 ~ 2872.5)	2475.0 (1822.5 ~ 3075.0)	< 0.001	1823.0 (1275.0 ~ 2423.0)	1950.0 (1485.0 ~ 2483.0)	0.340
Follicles ≥ 14 mm on hCG day, n	12.0 (8.0 ~ 15.0)	8.0 (6.0 ~ 10.0)	< 0.001	19.0 (16.0 ~ 22.0)	14.0 (11.0 ~ 17.0)	< 0.001
Peak E2(pg/ml)	2444.0 (1690.3 ~ 3707.3)	1804.5 (1271.3 ~ 2634.5)	< 0.001	4788.0 (3000.0 ~ 5991.0)	3310 (2403.0 ~ 4925.0)	0.002
P on hCG day(ng/ml)	0.8 (0.6 ~ 1.2)	0.7 (0.5 ~ 1.0)	< 0.001	1.0 (0.7 ~ 1.6)	0.9 (0.7 ~ 1.2)	0.068
Endometrial thickness on hCG day, mm	11.7 (10.0 ~ 13.6)	11.6 (9.8 ~ 13.4)	0.211	11.8 (10.4 ~ 12.8)	12.0 (10.4 ~ 13.8)	0.336
No. of oocytes retrieved, n	13.0 (9.0 ~ 17.0)	8.0 (6.0 ~ 10.0)	< 0.001	26 (22 ~ 29)	17 (14 ~ 21)	< 0.001
No. of mature oocytes, n						
Before IVM	2.0 (1.0 ~ 4.0)	6.0 (4.0 ~ 7.0)	< 0.001	10 (9 ~ 11)	13 (11 ~ 17)	< 0.001
After IVM	11.0 (8.0 ~ 14.0)	6.0 (4.0 ~ 7.0)	< 0.001	22 (21 ~ 27)	13(11 ~ 17)	< 0.001
Fertilization rate (%)	65.6	67.4	0.010	62.4	69.9	< 0.001
Blastocyst rate (%)	60.3	55.1	< 0.001	63.3	63.3	0.997
No available embryo, n	34 (3.9)	215 (12.5)	< 0.001	2 (5.1)	59 (2.2)	0.213

Values are expressed as median (minimum –maximum) for continuous variables and n (%) for categorical variables

**Table 4:** IVF/ICSI outcome characteristics of ICSI only and IVM with ICSI patients

	Mature oocytes < 9			Mature oocytes ≥ 9		
	IVM + ICSI (n = 873)	ICSI (n = 1725)	P	IVM + ICSI (n = 39)	ICSI (n = 2716)	P
**Fresh cycle**						
No. of fresh ET cycles	591	1225		7	1741	
Implantation rate (%)	410/838 (48.9)	753/1671 (45.1)	0.067	5/9 (55.6)	1199/2451 (48.9)	0.749
Clinical pregnancy rate, n (%)	333 (56.3)	629 (51.3)	0.046	4 (57.1)	1004 (57.7)	1.000
Abortion rate, n (%)	40 (12.0)	65 (10.3)	0.427	0	113 (11.3)	NA
LBR per ET, n (%)	293/591 (49.6)	564/1225 (46.0)	0.157	4 (57.1)	891 (51.2)	1.000
LBR per oocytes retrieval, n (%)	293/873 (33.6)	564/1725 (32.7)	0.657	4 (10.3)	891 (32.8)	0.003
**FET cycle**						
Cryopreservation rate, n (%)	692 (79.3)	958 (55.5)	< 0.001	35 (89.7)	2367 (87.2)	0.811
No. of freeze-all cycles	248 (28.4)	285 (16.5)	< 0.001	30 (76.9)	916 (33.7)	< 0.001
No. of FET cycles	606	717		39	2169	
Implantation rate	393/775 (50.7)	373/914 (40.8)	< 0.001	37/56 (66.1)	1712/3026 (56.6)	0.155
Clinical pregnancy rate, n (%)	337 (55.6)	335 (46.7)	0.001	28 (71.8)	1394 (64.3)	0.331
Abortion rate, n (%)	59 (17.5)	69 (20.6)	0.308	2 (7.1)	184 (13.2)	0.569
LBR per FET, n (%)	278/606 (45.9)	266/717 (37.1)	0.001	26 (66.7)	1210 (55.8)	0.175
LBR per oocytes retrieval, n (%)	278/873 (31.8)	266/1725 (15.4)	< 0.001	26 (66.7)	1210 (44.6)	0.006
**Cumulative outcomes**						
CPR per oocytes retrieval	616 (70.6)	932 (54.0)	< 0.001	32 (82.1)	2230 (82.1)	0.993
CLBR per oocytes retrieval	571 (65.4)	830 (48.1)	< 0.001	30 (76.9)	2101 (77.4)	0.949

To exclude the effect of other confounding factors, we performed binary logistic regression and inverse probability weighting (IPW) analysis (Table 5). The two models have similar conclusions. Rescue IVM had no significant effect on the live birth rate of fresh cycles in either group. However, performing Rescue IVM was a significantly positive factor in improving CLBR in patient with MII<9(aOR = 2.325 95%CI: 1.698–3.187, P < 0.001), but not in MII ≥ 9 group (aOR = 2.291 95%CI:0.922–5.693, P = 0.074).

**Table 5:** Odds ratios and adjusted odds ratios of pregnancy outcomes before and after binary logistic regression and IPW.

	Live birth	Crude Model^a^		Adjusted Model 1^b^	
	n (%)	OR (95%CI)	P value	Adjusted OR (95%CI)	P(a) value1
**Fresh cycles**					
**Mature oocytes < 9**					
IVM + ICSI (n = 591)	293 (49.6%)	1.152 (0.947 ~ 1.403)	0.157	1.274 (0.863 ~ 1.881)	0.222
ICSI (n = 1225)	564 (46.0%)	Reference		Reference	
**Mature oocytes ≥ 9**					
IVM + ICSI (n = 7)	4 (57.1%)	1.272 (0.284 ~ 5.700)	0.753	1.176 (0.243 ~ 5.699)	0.840
ICSI (n = 1741)	891 (51.2%)	Reference		Reference	
**Cumulative live birth**					
**Mature oocytes < 9**					
IVM + ICSI (n = 873)	571 (65.4%)	2.039 (1.723 ~ 2.413)	< 0.001	2.325 (1.698 ~ 3.187)	< 0.001
ICSI (n = 1725)	830 (48.1%)	Reference		Reference	
**Mature oocytes ≥ 9**					
IVM + ICSI (n = 39)	30 (76.9%)	0.976 (0.461 ~ 2.066)	0.949	2.291 (0.922 ~ 5.693)	0.074
ICSI (n = 2716)	2101 (77.4%)	Reference		Reference	

^a^ Covariates: age, infertility type, infertility duration, BMI, FSH, COH protocol, Gn duration, Gn dose, endometrial thickness, number of eggs retrieved, number of mature oocytes, type of transfer, No. of embryos transferred

^b^ Covariates: age, infertility type, infertility duration, BMI, FSH, COH protocol, Gn duration, Gn dose, number of eggs retrieved, number of mature oocytes

## Discussion

Our results show a significant positive relationship between the number of mature oocytes and cumulative live birth outcomes in first IVF/ICSI cycle patients, and find 9 and 11 as the appropriate cut-off point of the number of MII and total eggs for the CLBR respectively. Besides, based on a controlled analysis of patients with and without Rescue IVM, we conclude that Rescue IVM is effective in helping patients with a mature oocyte less than 9 to obtain more available mature oocytes, and resulting in better clinical outcomes.

Quality and quantity of the whole embryos are the two most important predictors of the cumulative outcome in IVF/ICSI [35]. With the total number of oocytes retrieved after COH, reproductive clinicians can describe the patients’ ovarian function accurately [27] and thus predict their IVF/ICSI live birth outcome. However, the role of immature oocytes is still very limited, the source of available embryos is mainly from mature oocytes. Moreover, for a subset of patients with oocyte maturation failure [36], the total number of eggs retrieved is more difficult to accurately describe their pregnancy outcome. Compared to previous studies, therefore, our study paid more attention to the effect of MII oocytes quantity on IVF/ICSI outcome, as reflected by the CLBR. Our result is generally similar to the overall tendency in previous studies about the total number of oocytes retrieved regarding fLBR or CLBR [16–18, 20–22]. Based on our data, the fLBR plateaued when the number of MII reached 8 and decreased after > 14. As for the CLBR per started cycle, the adjusted ORs for CLBR continued to increase with increasing MII without a plateau period. There are some differences in our study: firstly, when MII oocytes was used as the dependent variable instead of the total number of oocytes retrieved, the fLBR plateau appeared earlier (plateau in previous studies was mostly 12–18), it is a reasonable difference considering the average oocyte maturation rate of the total population in this study (88.12%). Another results in contrast with 2 single-center studies is that when using MII data as a basis for grouping, the group with higher numbers of mature oocytes had significantly higher oocyte fertilization rates, rather than decreasing with increasing number of oocytes retrieved [20, 22], a potential explanation for this difference is that after removing the effect of immature oocytes, the MIIoocytes retrieved from the patients with more MIIoocytes are of better quality. That result also explains why the number of mature oocytes would have a more significant effect on CLBR. On the one hand, patients with more MIIoocytes have better oocytes, and on the other hand, more quantities of MIIoocytes and embryos bring more opportunities to choose the better embryos for transfer. Results of the ROC curves also verify this result, that MIIoocytes has a stronger sensitivity and accuracy for CLBR.

Increasing the number of mature oocytes is the most obvious effect of Rescue IVM. Based on ROC results in the conventional IVF/ICSI patient, the cut-off point of mature oocytes for CLBR is 9. A previous retrospective study that included 737 cycles concluded that the minimum MII yield to predict live birth after ART was 6, and more than six mature oocytes did not result in a significant benefit in the take-home baby [37]. A reasonable explanation for the difference in cut-off values is due to the fact that this study only studied live birth rates in fresh embryo transfer and did not consider the situation in FETs. To verify our result, we further compared the treatment outcomes of patients who underwent ICSI only and ICSI with Rescue IVM, and found that for patients with MII counts < 9, performing Rescue IVM may help them achieve better clinical pregnancy rates and CLBR, whereas for patients who already have a high number of mature oocytes (≥ 9), Rescue IVM will not significantly improve their treatment outcome.

The large sample size and strict exclusion criteria are the strengths of this study. Despite the various methods we have used to reduce the generation of bias, we still need to acknowledge that this retrospective study has some limitations. Firstly, the strength of evidence from retrospective studies is weaker than other forms of research, such as prospective studies or randomized controlled trials. Secondly, the data is from a single center and considering the heterogeneity of reproductive hospitals in different regions, the conclusions may lack evidence of general application. Nevertheless, the conclusions of this study are still of guidance for clinical application as well as for further experimental investigations: from the aspect of clinical application, the number of MII oocytes is critical to achieving a satisfactory CLBR, suggesting that reproductive scientists should pay more attention to increase the oocyte maturation rate rather than simply increasing the number of oocytes retrieved through higher doses of gonadotropins. In addition, Rescue IVM is a potential rescue method for patients with a higher than expected percentage of immature oocytes after routine COH [4]. This study further clarifies the candidates for the application of Rescue IVM technique: patients with MII oocytes < 9 in a conventional IVF/ICSI cycle. In contrast, it is not necessary for patients who already have sufficient mature oocytes, thus avoiding over-medication. However, due to the limitations of the type of study, we hope that future large prospective or randomized controlled trials will further validate our conclusion.

In conclusion, the number of mature oocytes is an independent influence of CLBR in infertile patients undergoing IVF/ICSI treatment. After taking into account the frozen-thawed cycles, a higher number of MII tends to result in better cumulative live birth outcomes, and the appropriate cut-off point for MII oocyte count is 9. For the patients with less than 9 MII oocyte retrieved, the additional mature oocytes obtained by Rescue IVM contributed to better clinical outcome, in contrast, patients with MII oocyte ≥ 9 are not necessary for Rescue IVM.

### Electronic supplementary material

Below is the link to the electronic supplementary material.

Supplementary Material 1

## Data Availability

The data generated or analyzed during this study is available from the corresponding author upon reasonable request.
