# Supplementary material for: Cut-off point of mature oocyte for routine clinical application of rescue IVM: a retrospective cohort study
Source: J Ovarian Res. 2023 Nov 22;16:226. doi: 10.1186/s13048-023-01294-z (PMC10664607; doi:10.1186/s13048-023-01294-z)

**Supplementary Table 1.** Cut-off point of MⅡ oocytes and total oocytes for CLBR by Youden index

| **Criterion** | **Sensitivity [95% CI]** | **Specificity [95% CI]** | **Youden index** | **+LR** | **-LR** |
| --- | --- | --- | --- | --- | --- |
| **Mature oocytes** | | | | | |
| >7 | 77.95[77.3-78.6] | 58.94[57.8 - 60.1] | 0.3689 | 1.90 | 0.37 |
| ***>8*** | ***71.55[70.8-72.3]*** | ***65.61[64.5 - 66.7]*** | ***0.3716*** | ***2.08*** | ***0.43*** |
| >9 | 64.77[64.0-65.5] | 71.95[70.9 - 73.0] | 0.3672 | 2.31 | 0.49 |
| **Total oocytes** | | | | | |
| >9 | 73.02[72.3 - 73.7] | 61.85[60.7 - 63.0] | 0.3487 | 1.91 | 0.44 |
| ***>10*** | ***67.07[66.3 - 67.8]*** | ***67.93 [66.9 - 69.0]*** | ***0.3500*** | ***2.09*** | ***0.48*** |
| >11 | 60.90[60.1 - 61.7] | 72.75 [71.7 - 73.8] | 0.3365 | 2.23 | 0.54 |

**Supplementary Table 2.** Pairwise comparison of two ROC curves

| **Variable** | **AUC** | **SE** | **95% CI** | **Difference between areas** | **Z statistics** | **P-value** |
| --- | --- | --- | --- | --- | --- | --- |
| **Number of MⅡ** | 0.752 | 0.0035 | 0.746-0.758 | 0.0161 | 13.413 | ＜0.0001 |
| **Number of oocytes** | 0.736 | 0.0036 | 0.730-0.742 |  |  |  |

**Supplementary Figure 1.** Age subgroups of cumulative and fresh LBR based on the number of MⅡ oocytes retrieved. (A) age<30; (B) age:30-36(C) age>36


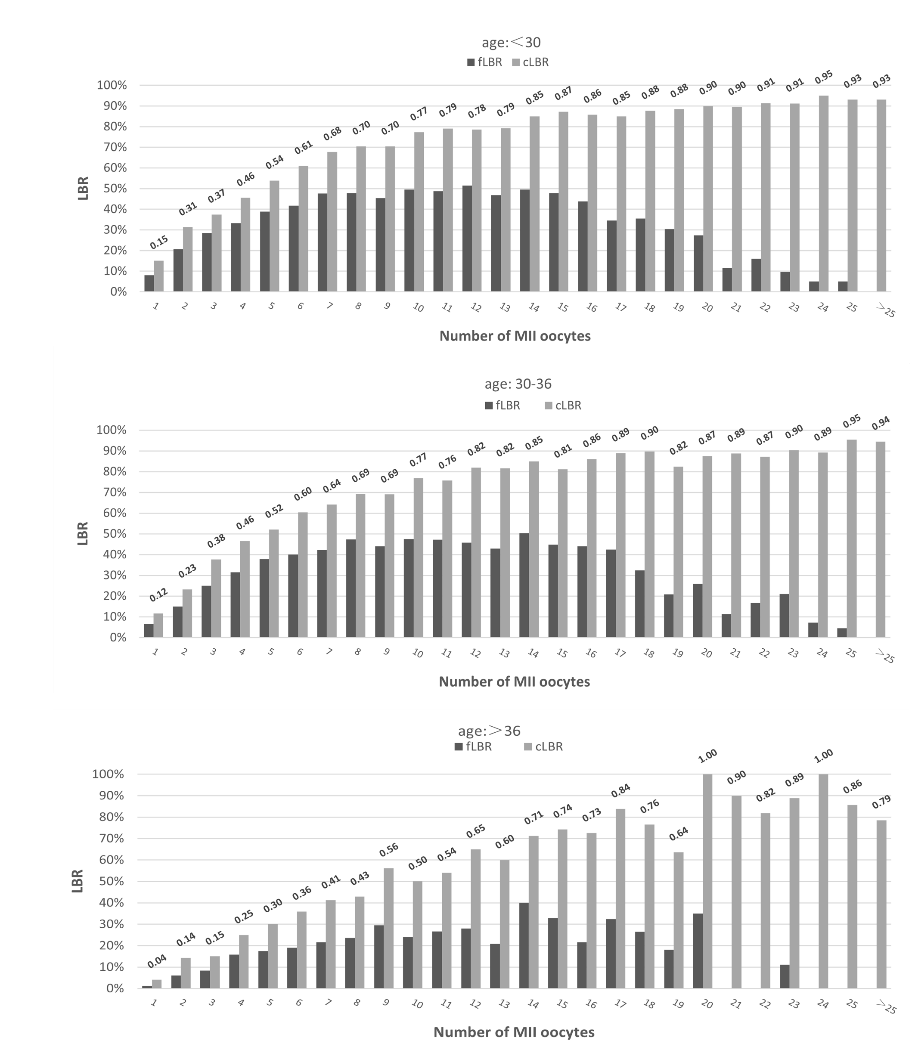


**Supplementary Figure 2.** The number of mature oocytes and total oocytes in the prediction of fLBR by receiver operator characteristic curve (ROC).


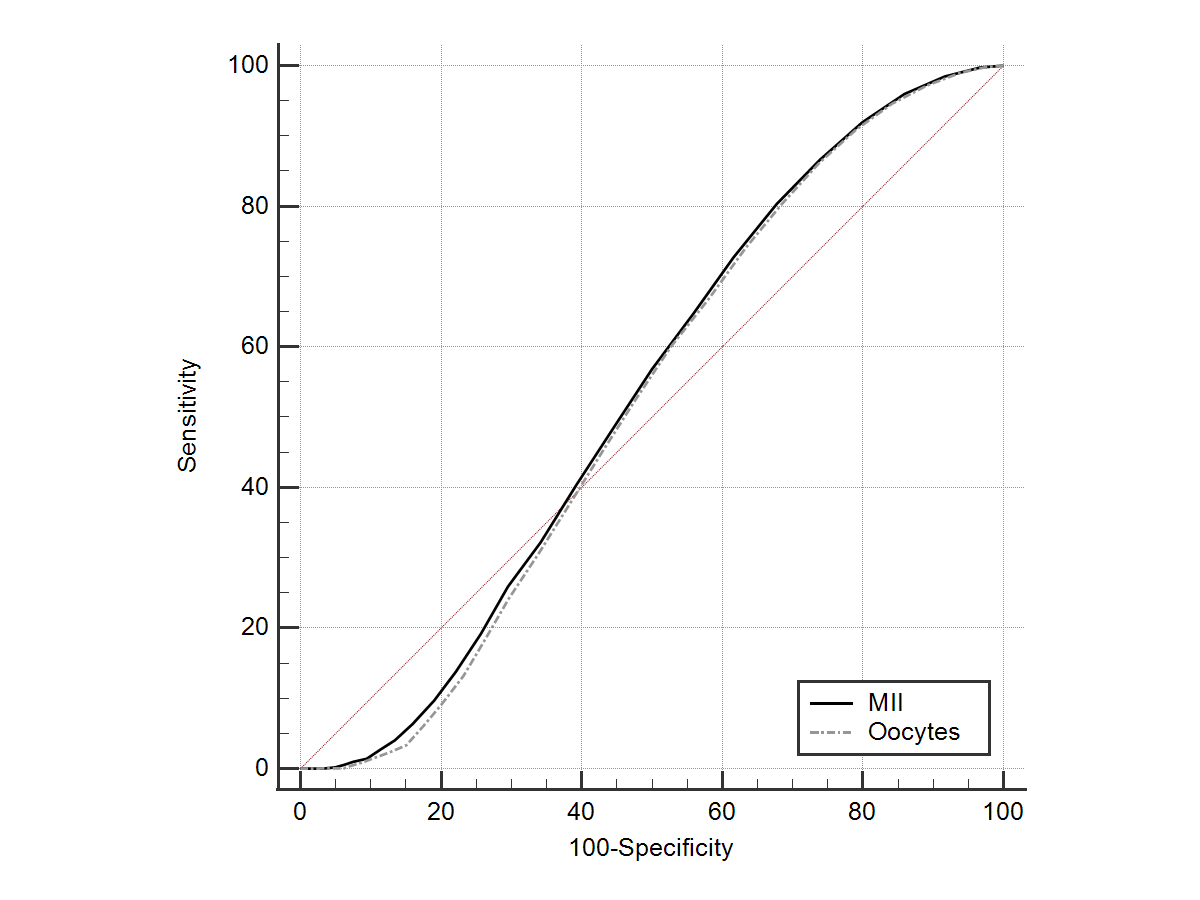

Supplement: Supplementary file 1 — Supplementary Material 1 [file 13048_2023_1294_MOESM1_ESM.docx]
